# Flow Diverter Performance Comparison of Different Wire Materials for Effective Intracranial Aneurysm Treatment

**DOI:** 10.3390/bioengineering11010076

**Published:** 2024-01-12

**Authors:** Yeo Jin Jun, Doo Kyung Hwang, Hee Sun Lee, Byung Moon Kim, Ki Dong Park

**Affiliations:** 1 Taewoong Medical, 14, Gojeong-ro, Wolgot-myeon, Gimpo-si 10022, Republic of Korea; yjjun@stent.net (Y.J.J.); dkhwang@stent.net (D.K.H.); hslee@stent.net (H.S.L.); 2 Department of Molecular Science and Technology, Ajou University, 206, World Cup-ro, Yeongtong-gu, Suwon-si 16499, Republic of Korea; 3 Department of Radiology, Severance Stroke Center, Severance Hospital, Yonsei University College of Medicine, 50-1, Yonsei-ro, Seodaemun-gu, Seoul 03722, Republic of Korea; bmoon21@yuhs.ac

**Keywords:** flow diverter, nitinol, platinum core nitinol, stent, aneurysm treatment, trackability force, simulated model

## Abstract

A flow diverter (FD) is an effective method for treating wide-necked intracranial aneurysms by inducing hemodynamic changes in aneurysms. However, the procedural technique remains challenging, and it is often not performed properly in many cases of deployment or placements. In this study, three types of FDs that changed the material of the wire were prepared within the same structure. Differences in physical properties, such as before and after delivery loading stent size, radial force, and radiopacity, were evaluated. The performances in terms of deployment and trackability force were also evaluated in a simulated model using these FDs. Furthermore, changes of deployment patterns when these FDs were applied to a 3D-printed aneurysm model were determined. The NiTi FD using only nitinol (NiTi) wire showed 100% size recovery and 42% to 45% metal coverage after loading. The low trackability force (10.9 to 22.9 gf) allows smooth movement within the delivery system. However, NiTi FD cannot be used in actual surgeries due to difficulties in X-ray identification. NiTi-Pt/W FD, a combination of NiTi wire and platinum/tungsten (Pt/W) wire, had the highest radiopacity and compression force (6.03 ± 0.29 gf) among the three FDs. However, it suffered from high trackability force (22.4 to 39.9 gf) and the end part braiding mesh tended to loosen easily, so the procedure became more challenging. The NiTi(Pt) FD using a platinum core nitinol (NiTi(Pt)) wire had similar trackability force (11.3 to 22.1 gf) to NiTi FD and uniform deployment, enhancing procedural convenience. However, concerns about low expansion force (1.79 ± 0.30 gf) and the potential for migration remained. This comparative analysis contributes to a comprehensive understanding of how different wire materials influence the performance of FDs. While this study is still in its early stages and requires further research, its development has the potential to guide clinicians and researchers in optimizing the selection and development of FDs for the effective treatment of intracranial aneurysms.

## 1. Introduction

Intracranial aneurysm (ICA) is an abnormal expansion of the cerebral artery wall. When it ruptures, it can lead to severe neurological impairment and a high mortality rate. Intracranial aneurysms are common acquired lesions that occur in 1–2% of the population [1,2] and account for about 80–85% of non-traumatic subarachnoid hemorrhages [3,4]. A meta-analysis of 33 studies showed a case fatality rate ranging from 8.3% to 66.7% in patients with subarachnoid hemorrhage [5,6].

Following the approval of the detachable micro coil, developed by Guido Guglielmi, by the United States Food and Drug Administration (FDA), endovascular coil embolization of cerebral aneurysms has spread worldwide [7,8,9,10]. Despite the rapid advancement in endovascular coil embolization therapy, large (≥20 mm) and giant (≥25 mm) intracranial aneurysms pose a significant challenge for both surgical and endovascular treatments. They are often associated with a high recurrence rate and an increased rate of procedural morbidity and mortality [11,12,13,14]. If left untreated, aneurysms, lesions that involve the intradural anterior cerebral circulation (internal carotid artery distribution) are associated with a 5-year cumulative risk of rupture of 14.5–40%, depending on the location [15,16]. Treatment of large and giant wide neck aneurysms with coils is associated with a low rate (~35%) of initial angiographic occlusion [17] and a high rate (>50%) of recurrence [18,19,20,21,22,23]. Even with the use of advanced coiling techniques (including adjunctive balloons, stents, and complex-shaped and biologically enhanced coils), improved outcomes remain elusive [24,25,26,27,28,29].

The flow diverter (FD) was developed for the intravascular treatment of such wide neck aneurysms by inducing hemodynamic changes in aneurysms. The concept of flow diversion therapy is that when a high metal density (low porosity) stent is placed across the aneurysm neck, it induces a change in the direction of blood flow away from the target aneurysm sac to the distal parent artery, leading to stasis of blood flow within the aneurysm. This stasis promotes intra aneurysmal thrombosis. The dense stent struts around the aneurysm neck can accelerate neointima formation [30].

FDs have evolved through the development of various products such as the Pipeline Flex Embolization Device (Medtronic, Minneapolis, MN, USA ), Silk Vista (Balt, Montmorency, France), Surpass Evolve (Stryker, Kalamazoo, MI, USA) and Fred System (Microvention, Aliso Viejo, CA, USA) [31,32,33,34]. Advancements in various FD products have improved the effectiveness of treating previously challenging or risky aneurysms. However, the procedural technique remains challenging, and it is often not performed properly in many cases of deployment or placements. Technical complications associated with FD malapposition and proximal migration may pose risks for delayed ischemic events or potentially delayed life-threatening aneurysm rupture [35,36,37].

Commercialized products have the same purpose of use, but their performance properties differ due to variations in materials and structures. To prevent risks during the procedure, a comprehensive understanding of the physical properties of the FD is essential. However, most current FD studies focus on clinical or hemodynamic flow, with very limited research exploring materials and physical characteristics. Considering the complexity of using FD, the procedural technique is very important [10]. Understanding the characteristics of the material and the performance properties derived from it can rapidly advance research for improving usability and procedural techniques.

Many FDs are manufactured using nitinol (NiTi) wire, a shape memory alloy, arranged in a braided mesh structure. It is a self-expandable stent because nitinol materials have superelastic properties under specific heat treatment conditions. However, nitinol has low density, which can result in limited visibility in X-ray imaging. Poor radiopacity makes it difficult to use intervention procedures. To solve this problem, radiopaque wire materials such as Pt/W, tantalum, and Pt core nitinol are used in combination with nitinol wire.

In this study, three types of FDs were prepared using nitinol, platinum/tungsten (Pt/W), and Pt core nitinol wire. Pt/W wire is an excellent radiopaque wire consisting of 8% tungsten and 92% platinum. Pt core nitinol wire is a material that has both the superelastic properties of nitinol, which is the outer sheath, and the radiopacity properties of the Pt core [38]. Key performance properties such as size before and after delivery loading, radiopacity, and radial force of these three types of FDs were then compared. In addition, we compared the trackability and deployment force performance of these FDs through simulated use in a vascular model and verified the deployment patterns in a 3D-printed internal carotid artery (ICA) aneurysm model. Through this study, we aim to determine how the physical properties of the same structure FD can change depending on the wire material and suggest a testing method for the development of high-performance FDs. 

## 2. Experimental

### 2.1. Flow Diverter Preparation

FDs production used 0.012” nitinol wire (Furukawa, Tokyo, Japan), 0.012” Pt/Tungsten wire (Johnson Matthey, London, England, UK), and 0.012” 30% Pt core nitinol wire (Fort Wayne Metals, Fort Wayne, IN, USA). Three types of test samples were prepared by changing the wire material. The nitinol flow diverter (NiTi FD) was constructed using 48 strands of NiTi wire. The nitinol-platinum/tungsten flow diverter (NiTi-Pt/W FD) was constructed using a mixture of 36 strands of NiTi wire and 12 strands of Pt/W wire. The Pt core nitinol flow diverter (NiTi(Pt) FD) was constructed using 48 strands of 30% Pt core NiTi wire. All samples were manufactured through braiding and heat-treating processes using stainless steel jigs and braiding machines (SJ481, Gana, Anyang-si, Republic of Korea),targeting specifications of 5.0 mm in diameter and 15 mm in length.

Prepared FDs were cut to target length, loaded into dedicated delivery systems, and used for testing. These FDs were attached to a flexible delivery wire, which had radiopaque end markers, and packaged in an introducer sheath. The stent-contact portion of the delivery wire was coated with silicon so that it could grip the FD, allowing for re-sheathing at any point prior to reaching 70% deployment [39].

### 2.2. Flow Diverter Size Variation

The outer diameter and the cell area between the mesh before and after loading the FDs into the delivery system were measured and compared. The outer diameter (×25) and cell area (×220) were measured at three locations (distal, center, and proximal) using a digital microscope (VITINY UM20, Microlinks, Kaohsiung, Taiwan). Metal coverage was calculated after measuring the wire length in the cell image [40].

### 2.3. Radiopacity

FDs were separated into plastic containers and placed in the X-ray image area of an angiography apparatus (Artis zee biplane, Siemens, Munich, Germany). The focal point was adjusted. The distance between specimens was about 22 cm. Images were obtained by irradiating X-rays under the same conditions used in the actual procedures (70 kV, 3.5 s, 62 mA).

### 2.4. Radial force

Flat plates were installed on the equipment of a Universal Test Machine (UTM, TO-102D, Testone, Siheung-si, Republic of Korea) and each sample was placed in the center of the lower flat plate. After 50% of FD’s unconstrained diameter was compressed and then expanded to the initial diameter, the radial force was measured with a 1 kg load cell (Testone, Siheung-si, Republic of Korea)). The compression and expansion forces were analyzed from raw data at the recommended blood vessel diameter (5.00 mm).

### 2.5. Trackability and Deployment Force

**Figure 1:**
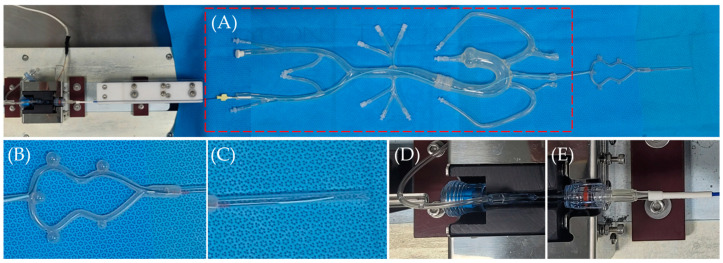
Trackability and deployment force test model. (**A**) Order-made aortoiliac bifurcation silicone vascular model (red dash box), (**B**) cerebral communicating artery silicone model, (**C**) 5.0 mm glass tube, (**D**) hemostatic valve, (**E**) microcatheter hub.

The model used had an in-house design. It has served as an analysis tool for various cerebral vascular stents, including FDs [41]. An order-made aortoiliac bifurcation silicone model (Figure 1A) was connected to the cerebral communicating artery silicone model (Figure 1B) and a 5.00 mm diameter glass tube (Figure 1C) was set at the end point. A 6Fr guiding catheter (Envoy^TM^, Cerenovus, Irvine, CA, USA) was inserted into the femoral artery of the silicone model. Through this, a 0.021-inch microcatheter (Prowler^®^ Select^TM^ plus, Cerenovus, Irvine, CA, USA) was inserted and passed through the vascular model to position the distal end of the microcatheter within the glass tube. A rotating hemostatic valve (RHV, Figure 1D) was connected to the hub of the microcatheter (Figure 1E).

After inserting the loader of the FD delivery system into the RHV, the valve was locked, and the RHV was fixed to the deployment force equipment (Gana, Anyang-si, Republic of Korea). Saline was injected through the side port of the RHV to flush the inside of the microcatheter and deploy it at a speed of 200 mm/min.

Continuous trackability force was recorded as the FD moved through the vascular model inside the microcatheter, and the deployment force applied to the glass tube was measured. The force at the 6-point position, where important changes occurred in the raw data, was compared.

### 2.6. Flow Diverter Deployment in a 3D-Printed ICA Aneurysm Model

The 3D-printed ICA aneurysm model is prepared using clear resin on a 3D printer (Form3, Formlabs, Somerville, MA, USA) based on the ICA aneurysm data. The printed model is cleaned and dried using isopropyl alcohol (IPA, Daejung Chemical, Siheung-si, Republic of Korea). The models are prepared at double the quantity, inspected for internal dimensions using a digital microscope (VITINY UM20, Microlinks, Kaohsiung, Taiwan), and then selected for use based on matching sizes.

**Figure 2:**
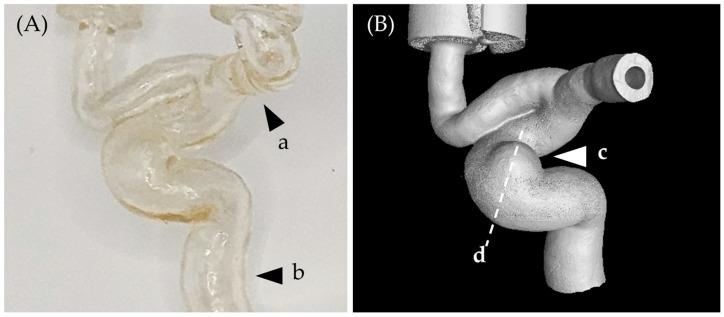
A 3D-printed ICA aneurysm model. (**A**) Optical image (a: distal end, b: proximal end), (**B**) micro CT image (c: ICA aneurysm, d: cross section location).

The microcatheter was inserted into the parent artery of a 3D-printed internal carotid artery (ICA) aneurysm model. The microcatheter tip was placed at the distal end (a in Figure 2) so that the FD could be deployed to the target point (a,b in Figure 2) with ICA aneurysm on the side. The delivery system was operated to deploy the FD inside the 3D-printed model to the proximal end (b in Figure 2). The same models were printed, and each FD was deployed in the same position.

Three-dimensional images of the deployed FD in the model were taken using a micro-CT (IVIS^®^ Spectrum CT, Perkin-Elmer, Shelton, CT, USA). The highest part of the ICA aneurysm (c in Figure 2) and the cross section (d in Figure 2) were measured, and the distance between the aneurysm and FD was compared.

### 2.7. Statistical Analysis

All measurements and experiments were conducted at least three times, and the data were expressed as mean ± standard deviation (s.d.). The data were analyzed using Microsoft Excel 2019 (Home and Business 2019, Microsoft Office, Redmond, WA, USA) and statistical significance was evaluated by a *p*-value of < 0.05.

## 3. Results & Discussion

### 3.1. Flow Diverter Stent Size

#### 3.1.1. Diameter Change before and after Delivery Loading

**Figure 3:**
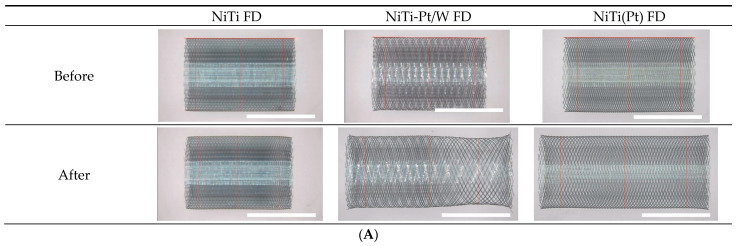

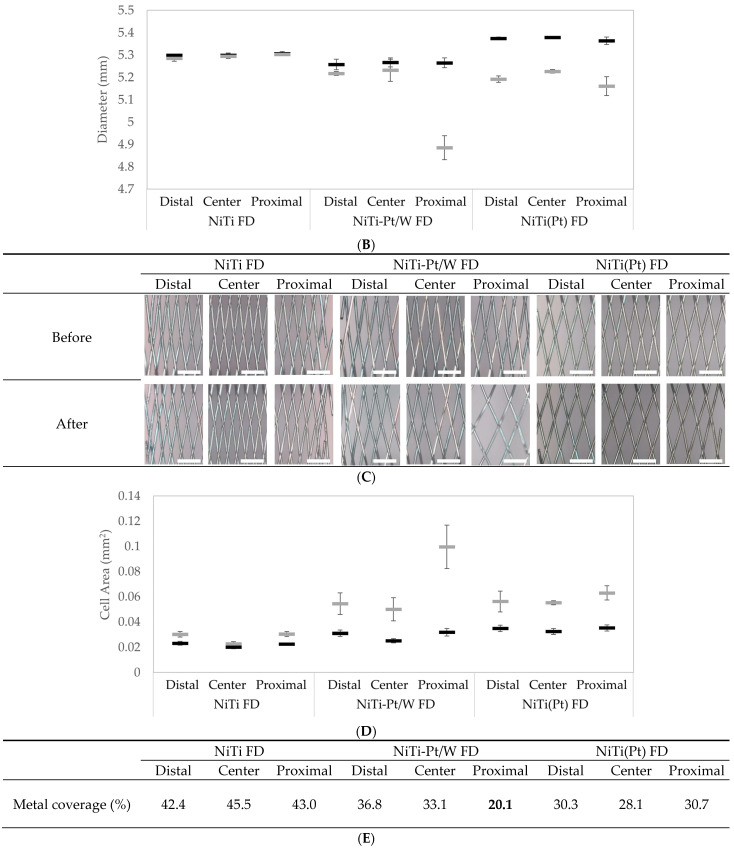
Flow diverter size change before and after delivery loading: (**A**) diameter size image (scale bar = 5 cm), (**B**) diameter size analysis (black bars: before, gray bars: after), (**C**) cell area image, scale bar = 0.3 mm, (**D**) diameter size analysis (black bars: before, gray bars: after), (**E**) metal coverage ratio after delivery loading.

An FD with a specification of 5 × 15 mm was manufactured as a target with an actual diameter of 5.25 mm [31]. Before loading, FDs were produced with a consistent diameter along the distal, center, and proximal regions (Figure 3A). Mean values for each were measured as follows: NiTi FD, 5.30 ± 0.01 mm; NiTi-Pt/W FD, 5.26 ± 0.02 mm; and NiTi(Pt) FD, 5.37 ± 0.01 mm (Figure 3B). NiTi-Pt/W was most similar to the target of 5.25 mm. The diameter increased in the order of NiTi-Pt/W FD, NiTi FD, and NiTi (Pt) FD. This was expected because the braiding and heat treatment conditions were optimized for NiTi-Pt/W. Even under the same manufacturing conditions, changes in wire material and composition might result in slight differences in sample size.

Since the FD was delivered to the procedure area using a microcatheter with a very small inner diameter of 0.021” (0.54 mm), it was used after being compressed to fit into a 0.54 mm tube during delivery loading. After delivery loading, it was observed that deployed FDs exhibited differences in diameter among the samples (Figure 3A). The NiTi FD, braided using a nitinol single wire with superelastic properties, almost fully restored its pre-loading diameter. In the case of the NiTi(Pt) FD, the change value seemed to be large since the diameter before loading was large. However, it decreased consistently overall and showed a difference of less than 0.1 mm from the target of 5.25 mm. When the NiTi-Pt/W FD was deployed after loading, the distal and center change values were less than 0.05 mm. However, the diameter differed by 0.38 mm in the proximal part (Figure 3B). This problem occurred when Pt/W wire, which lacked superelastic properties, constituted 25% of the entire FD composition. This is a common occurrence in FDs with a similar wire composition [31,42]. In particular, the proximal part is expected to change severely because it is loaded at the innermost part of the delivery system, experiencing the most friction and external force during the loading process.

#### 3.1.2. Cell Area Change before and after Delivery Loading

The FD cell area showed no significant differences among the distal, center, and proximal regions before loading (Figure 3C). Its average values for the NiTi FD, NiTi-Pt/W FD, and NiTi(Pt) FD were 0.022 ± 0.002 mm^2^ 0.029 ± 0.004 mm^2^, and 0.034 ± 0.003 mm^2^, respectively (Figure 3D). After delivery, loading, and deployment, there were differences in cell areas among the samples, similar to the differences in diameter.

The change value of the cell area was less than 0.01 mm^2^ for the NiTi FD. The cell area was almost restored before loading (Figure 3C). The metal coverage after loading was very high, ranging from 42% to 45%. For NiTi(Pt) FD, the change value in cell area was relatively large, ranging from 0.021 to 0.028 mm^2^. It changed consistently regardless of the location. The final metal coverage was from 28% to 30%. The metal coverage of 30% was similar to that of other products [43]. In the case of NiTi-Pt/W, the change value of the cell area was about 0.024 mm^2^ at both the distal and center positions, with good metal coverage, at 33–36%. However, the cell area was changed significantly in the proximal region, while the metal coverage remained at about 20% (Figure 3D,E).

Lower metal coverage can be disadvantageous for performance because metal coverage is related to the flow diversion effect. Achieving a high metal coverage is known to be a key factor in the success of stent-assisted aneurysm treatment [44].

### 3.2. Radiopacity

**Figure 4:**
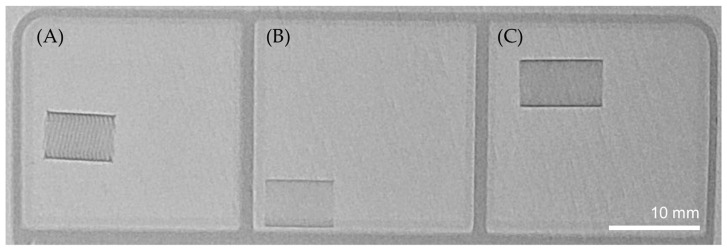
Flow diverter radiopacity image: (**A**) NiTi-Pt/W FD, (**B**) NiTi FD, (**C**) NiTi(Pt) FD.

The radiopacity image was captured under conditions similar to actual surgical procedures, except that it was taken from outside the human body, resulting in higher contrast measurements for the FD than in reality. The NiTi FD had the lowest visibility among the samples. It can be difficult to distinguish when the irradiation dose is low or when it is covered by thick tissues. The NiTi-Pt/W FD had the best visibility. The size and shape of cells were particularly well identified. In the case of the NiTi(Pt) FD, it was difficult to observe the detailed shape of the cell. However, the length, direction, and shape of the entire FD are clearly displayed (Figure 4).

### 3.3. Radial Force

**Figure 5:**
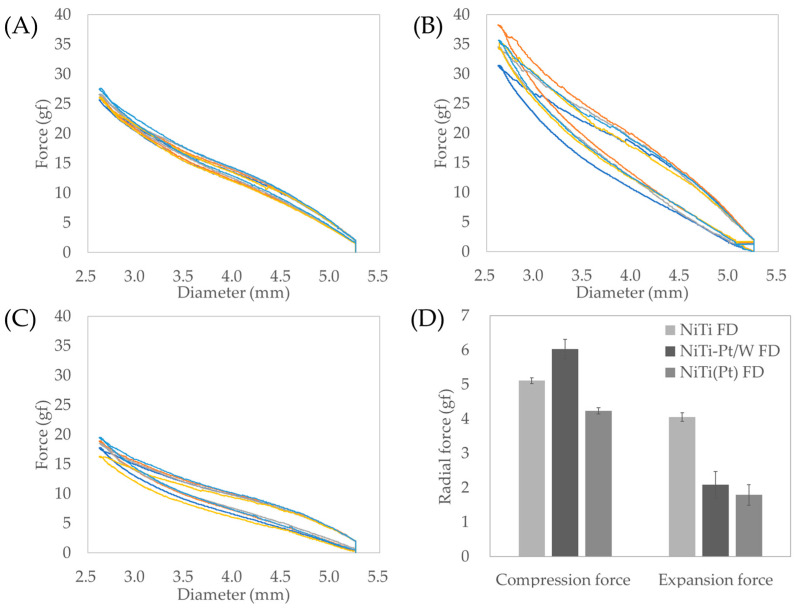
Flow diverter radial force: (**A**) NiTi FD, (**B**) NiTi-Pt/W FD, (**C**) NiTi(Pt) FD, (**D**) comparison of compression/expansion radial force at 5.00 mm point.

The NiTi-Pt/W FD had the highest compression force, at 6.03 ± 0.29 gf, followed by the NiTi FD (5.11 ± 0.08 gf) and the NiTi(Pt) FD (4.23 ± 0.09 gf). However, the NiTi FD had the highest expansion force, at 4.05 ± 0.12 gf, followed by the NiTi-Pt/W FD (2.09 ± 0.39 gf) and the NiTi(Pt) FD (1.79 ± 0.30 gf) (Figure 5D). The difference between compression force and expansion force is related to the restorative capability of the FD. With the smallest difference of −1.06 gf, the NiTi FD exhibited the most outstanding restorative capability, followed by the NiTi(Pt) FD (−2.44 gf) and the NiTi-Pt/W FD (−3.94 gf), indicating a progressive reduction in restorative performance.

The most insufficient restorative performance of NiTi-Pt/W FD was expected because Pt/Wire, which irreversibly changed due to the external force, affected the total FD expansion radial force. NiTi(Pt) FD can be considered to have an intermediate restorative capability between NiTi FD and NiTi-Pt/W FD. However, it had the lowest absolute radial force values. Thus, there might be problems such as migration.

### 3.4. Trackability and Deployment Force

**Figure 6:**
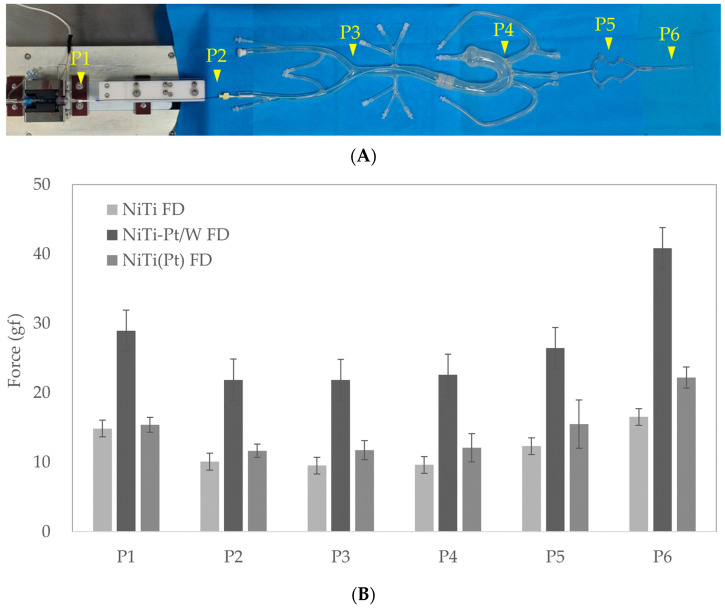
(**A**) Trackability force measurement points. P1: microcatheter insertion point, P2: guiding catheter starting point, P3: aorta starting point, P4: common carotid artery starting point, P5: curve simulated artery passing, P6: deployment force. (**B**) Flow diverter trackability and deployment force in a test model.

P1 was the section where the FD was inserted into the microcatheter. It had a high trackability force (from 15.5 to 31.1 gf) because there was a slight exposure to the outside between the delivery and the microcatheter hub. P2-P4 had a relatively lower trackability force (10.9 to 23.0 gf) because the microcatheter was maintained straight even in each transition section due to movement of the straight section within the blood vessel. In P5, the trackability force increased (14.6 to 27.4 gf) because friction occurred between the FD and the microcatheter due to movement in the curved vessel. P6 was the deployment section. The highest value (22.1 to 39.9 gf) was measured because there was an expansion force when the FD came out from the microcatheter (Figure 6). The overall trackability force was the highest for NiTi-Pt/W FD, ranging from 22.4 to 39.9 gf. NiTi FD and NiTi(Pt) FD had similar trackability forces, ranging from 10.9 to 22.9 gf and from 11.3 to 22.1 gf, respectively (Figure 6B). This result is expected to be related to the highest compression force of NiTi-Pt/W FD.

Since most aneurysm occurs in curved blood vessels, the actual deployment force is expected to be higher. A low deployment force makes it easier to fix the location of the microcatheter, making it advantageous for the FD to be placed at the desired location during the procedure.

### 3.5. Flow Diverter Deployment in 3D Printed ICA Aneurysm Model

Three different FD samples were deployed in the same location of a 3D printed ICA aneurysm model. All three were successfully implanted to the parent artery with a wide-neck aneurysm.

**Figure 7:**
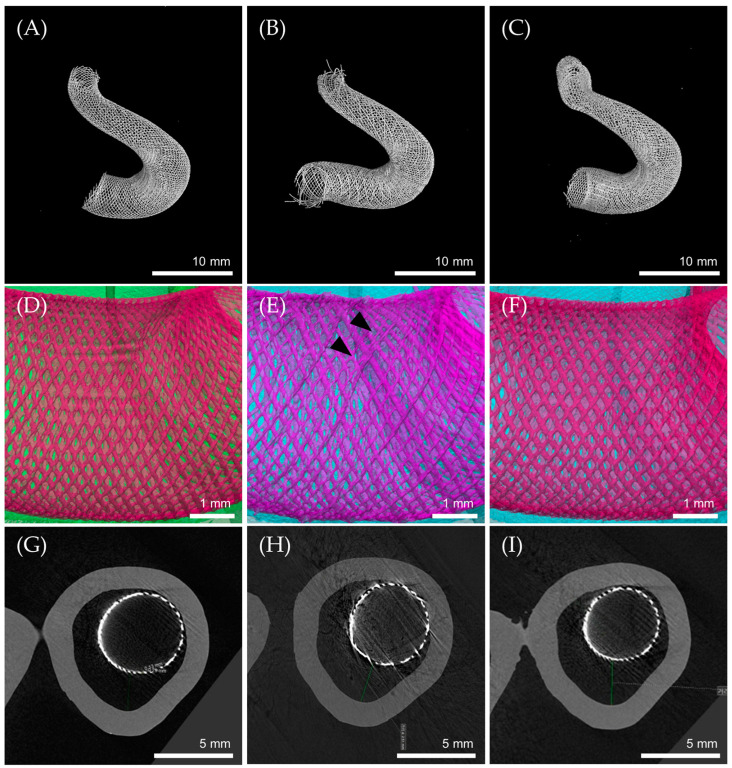
Micro CT image (**A**–**C**): flow diverter CT image, (**D**–**F**): wire arrangement, (**G**–**I**): aneurysm cross-section, (**A**,**D**,**G**): NiTi FD, (**B**,**E**,**H**): NiTi-Pt/W FD, (**C**,**F**,**I**): NiTi(Pt) FD.

The NiTi FD was deployed with an excellent radial shape at the desired location (Figure 7A). In the case of the NiTi-Pt/W FD, the distal position was well fixed. However, the proximal end was deployed closer than the desired point. This was expected to be a problem caused by pulling the FD together when removing the microcatheter due to high deployment forces. Furthermore, the end part of the NiTi-Pt/W FD braiding mesh became loose. This was expected because braiding weakened the binding force due to the repetitive friction during deployment and the heterogenous properties of NiTi-Pt/W (Figure 7B).

On the contrary, the NiTi(Pt) FD was anchored at the desired proximal location. However, the distal end was deployed further than the desired point. Due to the low radial force of the NiTi(Pt) FD, it was expected that the distal end would not be fixed well in the model. It migrated during delivery (Figure 7C). In all FD samples, the cell size remained consistent. However, in the case of the NiTi-Pt/W FD, it was observed that the arrangement of wires was misaligned due to heterogeneity arising from the mixing of NiTi wire and Pt/W wire (Figure 7E, black arrows). Such misalignment might induce specific weaknesses in FDs, which could potentially result in the development of kinks.

In the cross section of the ICA aneurysm, the distance between aneurysm and stent increased in the following order: NiTi FD, NiTi-Pt/W FD, and NiTi(Pt) FD (Figure 7G–I). The NiTi FD with the best restorative performance had the smallest gap (2.32 mm) with the model surface. The NiTi-Pt/W FD (2.51 mm) and the NiTi(Pt) FD (2.81 mm) were expected to move during delivery, resulting in an increased gap. However, the 3D printed ICA aneurysm model used is different from actual use because it is made of a hard and frictionless material. For a more accurate model evaluation, a 3D-printed model made of a material similar to a blood vessel is required.

### 3.6. Limitations and Future Work

The differences in properties due to material changes can impact the usage of FDs. Therefore, this research could be beneficial for the usability of FDs in clinical applications. However, being at a foundational stage, this study has certain limitations and lays the groundwork for future research.

Firstly, the characteristics of FDs are influenced not only by the material but also by factors such as structure, heat treatment methods, and delivery systems. In this study, we utilized a self-developed structure, equipment, method, and delivery system, so the performance may not be directly comparable to other commercialized products. All results should be considered with a focus on related materials. Secondly, the test method was designed to mimic real clinical conditions, but some test procedures were simplified for standardization of evaluation. Thirdly, ICA aneurysm models exhibit a wide range of structures, and it is challenging to consider the model used in this study as representative for evaluating the performance of FDs. The structure and size of an ICA aneurysm vary widely, and there is limited information on the necessary FD characteristics for different target aneurysms. We plan to apply 3D printed ICA aneurysm models with more diverse structures and gather additional information through animal experiments.

## 4. Conclusions

A flow diverter was developed for intravascular treatment of wide neck aneurysms by inducing hemodynamic changes within the aneurysms. This approach is effective in treating aneurysms that are difficult to treat using coil embolization. Throughout years of development, we have acquired an FD manufacturing system and are continuously improving it. Based on this manufacturing system, this study produced three different types of FDs, using NiTi, Pt/W, and Pt core nitinol wires, and compared their performance properties.

The NiTi FD has an excellent size recovery, a high metal coverage, and a low trackability force. It has many advantages in terms of performance and usability. However, it cannot be used in actual procedures due to difficulties in X-ray identification. To enhance its radiopacity, radiopaque wire materials such as Pt/W, tantalum, and Pt core nitinol can be used and combined with NiTi wire.

The NiTi-Pt/W FD, which improves radiopacity by incorporating Pt/W wire, has the advantages of high compression force and radiopacity. However, there is the problem of increased procedural difficulty due to the high trackability force. In addition, the end part braiding mesh tends to loosen easily. This issue is attributed to the combination of wires with different physical properties.

The NiTi (Pt) FD made of Pt core nitinol wire offers enhanced procedural convenience due to its low trackability force and uniform deployment. Nevertheless, concerns persist regarding potential migration due to the low expansion force.

In this study, three flow diverters were prepared with different wire types or combinations: NiTi FD, NiTi-Pt/W FD, and NiTi(Pt) FD. Their physical properties were compared. It was verified that the performance of the three types of FDs had complementary advantages and disadvantages. Excluding the NiTi FD, which is challenging to use in actual surgeries due to radiopacity issues, both the NiTi-Pt/W FD and the NiTi(Pt) FD are expected to have their different advantages, making them suitable for different clinical applications. However, the structure and size of ICA aneurysms vary widely, and there is a lack of research on which FD properties are necessary in different surgical situations. We plan to apply more diverse structures of 3D-printed ICA aneurysm models and gather additional information through animal experiments.

This comparative analysis contributes to an overall understanding of how different wire materials influence the performance of FDs. The findings derived from this study can guide clinicians and researchers in optimizing the selection and development of FDs for the effective treatment of intracranial aneurysms.

## Data Availability

The data supporting reported results are available from the corresponding author upon reasonable request.
